# Comparative analysis of the genome sequences and replication profiles of chikungunya virus isolates within the East, Central and South African (ECSA) lineage

**DOI:** 10.1186/1743-422X-10-169

**Published:** 2013-05-30

**Authors:** Karen Caiyun Chen, Yiu-Wing Kam, Raymond Tzer Pin Lin, Mary Mah-Lee Ng, Lisa FP Ng, Justin Jang Hann Chu

**Affiliations:** 1 Department of Microbiology, Laboratory of Molecular RNA Virology and Antiviral Strategies, Yong Loo Lin School of Medicine, National University Health System, National University of Singapore, MD4, 5 Science Drive 2, Singapore 117597, Singapore; 2 Singapore Immunology Network, Agency for Science, Technology and Research (A* STAR), 8A Biomedical Grove, #04-06, Immunos, Singapore 138648, Singapore; 3 National Public Health Laboratory, Communicable Disease Division, Ministry of Health, College of Medicine Building, 16 College Road, Singapore 169854, Singapore; 4 Department of Microbiology, Flavivirology Laboratory, Yong Loo Lin School of Medicine, National University Health System, MD4, 5 Science Drive 2, National University of Singapore, Singapore 117597, Singapore

**Keywords:** Chikungunya virus, Genome sequencing, Virus morphogenesis, Virus replication kinetics

## Abstract

**Background:**

A comparative analysis of the genomic and replication profiles of different geographical chikungunya virus (CHIKV) isolates of the East, Central and South African (ECSA) lineage was performed.

**Findings:**

Analysis of the data revealed the different growth kinetics for the different isolates. Deep genome sequencing analysis further revealed specific amino acid mutations in the viral nsP1, nsP3, nsP4, E1 and E2 proteins in the different isolates. Despite the difference in viral genomic profiles, the virus-induced ultrastructural changes within infected cells remained highly conserved among the different chikungunya virus isolates.

**Conclusions:**

These findings provide insights into the genomic and replication profiles of the re-emerging chikungunya virus isolates of the ECSA lineage.

## Findings

Chikungunya virus (CHIKV) was first isolated following an outbreak in Tanzania in the 1950s [1]. CHIKV infection typically induced an acute onset of painful syndrome as characterised by polyarthralgia, fever, asthenia, headache, myalgia and skin rashes [2,3].

CHIKV is an enveloped virus that possesses a single-stranded, positive-sense RNA genome. The 12Kb viral genome begins with an UTR at the 5’-terminal and followed by two-third of the coding regions for the non-structural proteins (nsP1-4), and the remaining one-third of the coding regions encodes for the structural proteins (Capsid-E3-E2-6K-E1), and a 3’-terminal poly-A-tail [4]. The non-structural proteins are processed into four nsPs and responsible for different roles in virus replication. nsP1 is important in viral RNA synthesis initiation and viral RNA capping [5]. nsP2 possesses protease and RNA helicase activities [6,7]. nsP3 is composed of three domains and this viral protein is required for the formation and localization of replication complexes [7,8]. nsP4 possesses RNA-dependent RNA polymerase (RdRp) activity that is important for replication and synthesis of the viral genome [8,9].

The urban transmission of the CHIKV involves the naive human host and the *Aedes* mosquito, with *Aedes aegypti* and *Aedes albopictus* as the main vectors [10]. Although the mortality rate is rather low, the epidemic potential of the virus is rather fearful due to the prolonged morbidity and the rapid spread of the virus. In 2004, the virus managed to catch fresh attention when it re-emerged and set its foot around the world. The first wave started in Kenya, the re-emergence of the virus was then reported in countries around the Indian Ocean [11,12]. From 2006 onwards, more countries including India and Sri Lanka have also reported severe outbreaks of the CHIKV infections [13]. In addition, outbreaks of CHIKV were also documented in South East Asia, with countries such as Malaysia, Thailand and Singapore being affected [11].

Phylogenetic analysis of CHIKV genome revealed three lineages namely: Asian, West African (WA) and East, Central and South African (ECSA) [14]. Viral genome sequence analysis revealed that the CHIKV isolates from the recent outbreaks in the India Ocean and South East Asia were predominantly attributed by the ECSA lineage [12,15,16]. In addition, detailed phylogenetic analysis further identified two distinct sub-lineages in the ECSA clades between the outbreaks in Indian Ocean island and India [11]. The CHIKV outbreak in Indian Ocean, in particularly island of La Reunion, carries a distinct substitution of alanine for valine at the 226 position of the CHIKV E1 protein. This E1:A226V substitution has been suggested to allows a better adaptation of the virus in the *Aedes albopictus* as compared to classical vector, *Aedes aegypti,* which leads to a rapid dissemination of the virus in La Reunion [17]. In addition, the re-emergence CHIKV outbreak occurring in the South East Asia was determined to be more related to the Indian sub-lineage of ECSA as compared to the Indian Ocean [12].

Singapore had it first outbreak with CHIKV in the year of 2008 [18,19]. In 2008 alone, several outbreaks have occurred in Singapore with the first outbreak occurring in January 2008 and genome sequencing of the CHIKV isolate revealed that the virus does not harbour the distinct E1:A226V mutation. The subsequent episodes of outbreaks in Singapore were caused by the CHIKV with the distinct E1:A226V mutation [18]. Here, in this study, we are interested in the comparative analysis of the genomic sequences and replication profiles of the different Singapore CHIKV isolates and the La Reunion (IMT) CHIKV isolate that are belonging to ECSA lineage. The first CHIKV isolate, SGP007 is a wild type E1:226A CHIKV isolated from patient in Singapore in 2008. The second CHIKV isolate SGP011, contains the E1:A226V mutation and was also isolated from patient during the Singapore outbreak in 2008 [20]. The third isolate, La Reunion (IMT) was isolated from the outbreak in La Reunion that contains the E1:A226V mutation [20]. All three isolates used were of low passage numbers upon isolation from patients.

**Figure 1:**
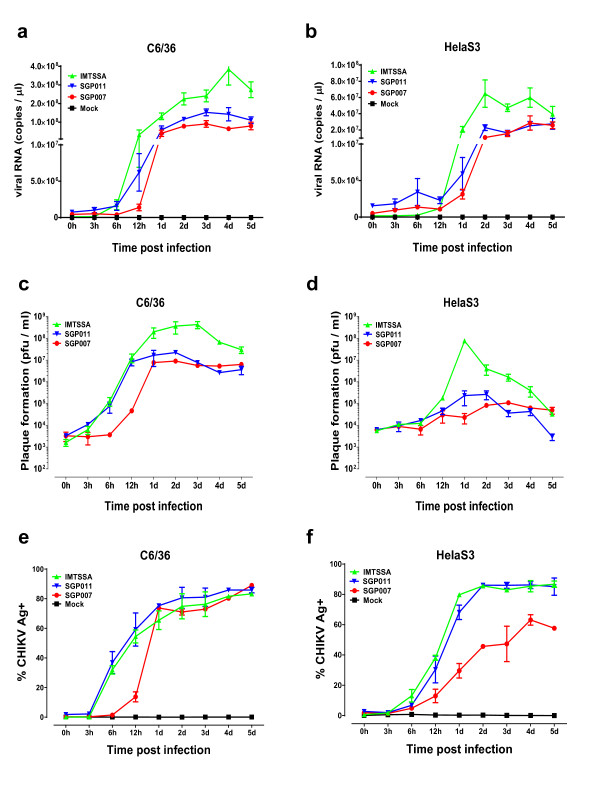
**Replication kinetics of CHIKV isolates (IMTSSA, SGP011 and SGP007) in infected C6/36 and HeLa cells. a** and **b**) Negative strands viral RNA quantification with qRT-PCR of CHIKV infected C6/36 and HeLa cells over a period of five days post-infection. **c** and **d**) Infectious virion formation from CHIKV infected C6/36 and HeLa cells were analyzed by plaque formation assay over a period of five days post-infection and expressed as log PFU/ml. **e** and **f**) FACS quantification of CHIKV antigen in infected C6/36 and HeLa cells over a period of five days post-infection.

In the first part of the study, the virus infection was performed at a multiplicity of infection (MOI) of 10 and the replication kinetics of the three CHIKV isolates (SGP007, SGP011 and IMT) was analysed by negative-strand viral RNA production via quantitative RT-PCR (qRT-PCR) within *Aedes albopictus* (C6/36) cells and HeLa cells. Viral RNA was extracted from CHIKV-infected cell cultures using QIAamp viral RNA MINI kit (Qiagen, Hilden, Germany) and the qRT-PCR was performed using QuantiTect® Probe RT-PCR Kit (Qiagen, Hilden, Germany) with Applied Biosystems (ABI) 7900HT Fast Real-Time PCR System, using a TaqMan assay modified from Plaskon and colleagues [21]. In the C6/36 cells, we observed three distinct rate of growth kinetics for the three different CHIKV isolates. The E1:A226V substitution in the E1 glycoprotein of the La Reunion isolate (IMT) allowed better adaptation in the C6/36 cells and resulted in a rapid increase in viral RNA copies as early as 6 hours post infection. The Singapore isolates (SGP007 and SGP011) started increasing in viral RNA copies from 12 hours post-infection. The SGP011 isolate, containing the E1:A226V substitution in the E1 glycoprotein, showed higher copies of viral RNA when compared to the SGP007 isolate, but still showed a poorer adaptability compared to the IMT isolate (Figure 1a). We then compared the replication kinetics in HeLa cells, a susceptible human cell line. Generally, we saw the same trends as for the C6/36 cells, with a sharp increase in viral load in the IMT isolate when compared to the two Singapore isolates (Figure 1b). Interestingly, it was noted that the infectious virus titres obtained by viral plaque assays further supported the notion that the IMT isolate produced a much higher infectious virus titre in both C6/36 and HeLa cells (Figure 1c and d) when compared to both SGP007 and SGP011 isolates. Notable, it was observed that SGP011 produced infectious viruses at an earlier time point of 12 hours post-infection when compared to SGP007 in C6/36 cells.

We then proceeded to detect the presence of CHIKV antigens using flow cytometry in an effort to further define the virus replication kinetics. CHIKV-infected cells were fixed and permeabilised (BD Bioscience) before being stained with an anti-alphavirus Ab (10 μg/ml, Santa Cruz) for 30 min, followed by APC-conjugated goat anti-mouse IgG F(ab)2 Ab (10 μg/ml, Invitrogen) for 30 min. Data was acquired in BD FACS Canto II (BD Biosciences) using BD FACSDiva software. In both C6/36 and HeLa cell lines, IMT and SGP011 isolates demonstrated similar infectivity kinetics and both isolates showed a significantly higher viral antigen staining compared to SGP007 (Figure 1e and f). This suggested that both IMT and SGP011 exhibited higher virus infectivity and viral antigen production in the infected cells when compared to SGP007 isolate.

To better understand if the differences in replication kinetics of the different CHIKV isolates were associated with specific mutation(s) in the viral genomes, viral RNA was isolated from each CHIKV isolate and reverse-transcribed into cDNA and sent for sequencing. The three cDNA libraries were sequenced as a multiplex in a single lane using the Illumina HiSeq2000 (Next Generation Sequencing Core facility, Genomic Institute of Singapore) at a coverage of 67436×, representing about 20 million reads per sample. *De novo* assembly and mapping of each sequence was performed using the velvet assembler and bowtie enabled by Pipeline Pilot, with hash size = 31 and insert size = 150. All three sequences seemed to be missing 20–50 bp from the 3' and 5' ends when compared to the prototype isolate (S27 African prototype). In order to perform end-repair, 100 000 reads were mapped with bowtie to the prototype isolate, and some of the end sequences were recovered. The libraries were compared with the ERCC spike-in RNA controls (Invitrogen), and all three libraries had a > 0.99 correlation between normalised read counts and the control concentrations in a log scale.

When compared to the CHIKV S27 African prototype isolate [22], we identified seven nucleotide (nt) substitutions within the genome of the SGP007 CHIKV isolate that resulted in amino acid (aa) changes (Additional file 1: Table S1). S27 African prototype and SGP007 CHIKV isolates had identical amino acid residues at various positions (nsP3 aa524, nsP4 aa75 and E1 aa226). The E1 A226V mutation suggested a closer identity between these two isolates. However, SGP007 CHIKV obtained an extra unique mutation at various positions (nsP1 aa60, nsP3 aa137, nsP4 aa563 and E2 aa178). It remains unknown whether these mutations will support the unique feature of SGP007 CHIKV in terms of virus replication. We did not identify any unique mutations within the nsP2, Capsid, E3 or 6K protein sequences.

Till now, there is limited information available regarding the relationship between the unique amino acid residues and the functions of alphavirus nsPs. Previous studies have suggested the role of E1 A226V mutation in the fitness of CHIKV in *Aedes aegypti* and *Aedes albopictus* mosquitoes [23]. Further studies have also suggested the role of E2 L210Q mutation in the sequential adaptive mutations in CHIKV and enhancement of efficient vector switching in nature [24]. More amino acid positions within the CHIKV structural proteins have been demonstrated to show positive selection effect in virus circulation and persistence in endemic areas [25]. Here, we would like to postulate the role and effect of amino acid mutations in the nsPs on virus replication efficiency.

We first identified one unique mutation in SGP007 (nsP3 A137V) located within the macro domain region (Additional file 1: Table S1). The first 160 amino acids of the N-terminal region of nsP3 are known as the macro domain, which possesses ADP-ribose 1”-phosphate phosphatase activity. In addition, a study using Sindbis virus suggested an important role of the nsP3 macro domain in virus replication [26]. However, structural mapping suggested that this amino acid is not positioned within the enzymatic groove of the protein (data not shown). Therefore, it is unlikely that mutations at this position would alter the phosphatase activity and further studies would be needed to investigate the importance of this mutation (nsP3 A137V) in CHIKV virus replication.

We further suggested a possible role for the *opal* stop codon that located at the C-terminal of the nsP3 proteins in enhancing CHIKV virus replication *in vitro*. Several alphaviruses express two different isoforms of nsP3, depending on the presence of an *opal* stop codon or an open reading frame upstream of nsP4 (7 amino acids before the C-terminal of the nsP3) [27,28]. Expression of CHIKV nsP3 in different isoforms with different properties or functions remains to be further characterized. CHIKV S27 African prototype and SGP007 have an identical amino acid residue at nsP3 position 524 (arginine), but in IMT and SGP011, this position encodes a stop codon instead. *In vitro* infection results showed that SGP007 resulted in lower virus replication efficiency when compared to IMT and SGP011 (Figure 1). It was shown previously that the *Opal* stop codon at this particular position improved O’Nyong-Nyong virus infectivity, relative to viruses encoding an arginine at the corresponding position [29]. In addition, a study using Semliki Forest virus also showed improved nsP3 protein stability after removing the nsP3 C-terminal region [28]. Therefore, our results are consistent with previous findings from other alphaviruses that CHIKV isolates possessing an opal stop codon upstream of nsP4 could improve nsP3 protein stability and virus infectivity. This in turn could enhance virus replication. In addition, the T75A amino acid residue at the nsP4 N-terminal region was found to correlate with the nsP3 C-terminal region in regulating CHIKV viral replication.

One last unique mutation identified from CHIKV SGP007 was the I563T amino acid residue at nsP4 located within the RdRp region. Within the structural region, one unique mutation identified from CHIKV SGP007 was the R178H amino acid residue at E2 glycoprotein and is located within the E2 domain B region. However, the precise role of the differences in these amino acids in the various CHIKV isolates in virus replication remains undefined.

To further address the issues if the specific amino acids differences between the different CHIKV isolates will affect the formation and morphogenesis of virus-induced structures during CHIKV replication within cells, we utilised transmission electron microscope (TEM) as a tool to decipher these replication processes within the infected cells. The replication process of CHIKV has not been well-defined and many studies are still based on the assumption on other established alphaviruses. In this part of the study, the C6/36 cells were infected with CHIKV at the MOI of 10 and morphological changes were observed and documented under TEM at different time points post-infection. Similarly, the HeLa cells were infected with CHIKV at the same MOI and processed for the ultrastructural analysis at different time points post-infection with the different isolates of CHIKV.

**Figure 2:**
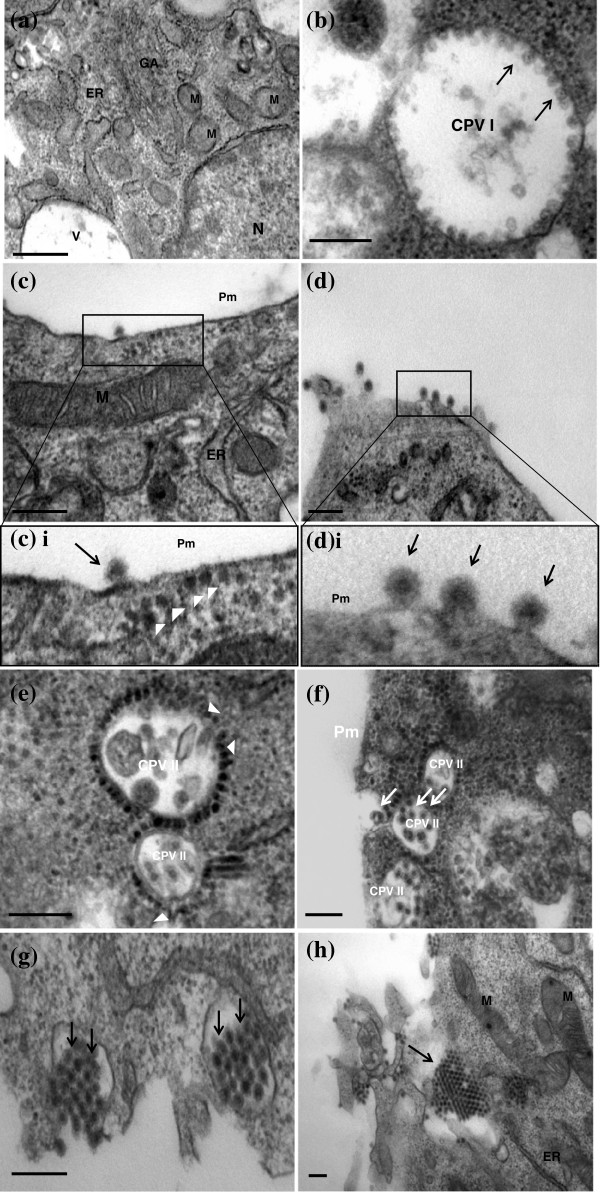
**Replication of CHIKV (IMT isolate) in C6/36 cells.** (**a**) Ultrastructural analysis of mock-infected C6/36 cells. At higher magnification, Golgi apparatus (GA), endoplasmic reticulum (ER) and mitochondria (M), can be observed. The bar corresponds to 0.5 μm. (**b**) Presence of Cytopathic Vacuoles (CPV) I in early infection (8 h post-infection). A typical CPV I complex (appx 800 nm) can be observed. CPV I is characterized by membranous spherules (arrows) regularly arranged from the interior surface of the complex. The bar corresponds to 0.2 μm. (**c**) Virus release by budding at the plasma membrane during early infection. CHIKV particle are observed to be budding from the plasma membrane (Pm). The bar corresponds to 0.2 μm. (**c**) i Enlarged of (**c**) showing the lining of the nucleocapsids (arrowheads) underneath the plasma membrane (Pm) of the cell. Matured virus (arrow) is observed to be budding from the peripheral of cells membrane. (**d**) Budding of CHIKV occurred extensively at the plasma membrane of the cells. The bar corresponds to 0.2 μm. (**d**) i Enlarged of (**d**) monitored the different stages of budding of CHIKV particles from the plasma membrane (arrows). (**e**) Ultrastructural analysis of late infection with CHIKV in C6/36 cells (Day 1 post infection). CPV II formations are observed. The viral nucleocapsids (arrowheads) are present at the cytoplasmic surface of CPV II complexes. The bar corresponds to 0.2 μm. (**f**) Several CPV II complexes are found in cluster near the plasma membrane (Pm) with the noticed of mature virus (arrows) in the complex. (**g**) and (**h**) At the late stage, CHIKV can be seen to be released from cells via exocytosis (arrows). Both the bar of g and h correspond to 0.2 μm.

In the mocked-infected cells, normal morphology of the cells was observed and recorded (Figure 2a). The C6/36 cells were characterized by large nuclei, occupying two-thirds of the cells and the other cellular organelles were also observed. At higher magnification, stacks of Golgi apparatus, smooth and rough endoplasmic reticular and mitochondria were clearly identified and documented (Figure 2a).

Generally, upon infection with the different CHIKV isolates, the virus induced structures and morphogenesis observed were highly conserved. The first distinct viral-induced structures observed, were the presence of the cytopathic vacuoles type I (CPV I) complexes. This occurred as early as 6 to 8 hours post-infection. These distinct vacuoles measured to be approximately 800 nm to 2000 nm in diameter and are characterized by the presence of membranous spheres lining on the vacuoles (Figure 2b). These membranous spheres (Figure 2b, arrows) measured to be 60 nm to 80 nm in sizes and were seen to be neatly arranged on the vacuoles. These vacuoles were also documented in cells infected with other alphaviruses and these vacuoles were proven to be the replication complexes where viral RNA synthesis occur and were seen in the early infection [30]. Other studies also showed the presence of the viral replicases in CPV I complexes of other alphaviruses. The staining of the cellular markers for the cytoplasmic endosome and lysosome suggested the origin of these CPV I complexes [31,32]. As the infection progressed to 10 hours post-infection, the degenerated forms of CPV I can be observed. The membranous spheres within the CPV I complexes are no longer visible and are replaced by vacuoles containing bags of membranous whorls (data not shown).

The appearance of the CPV I complex at 8 hours post-infection was accompanied by the emergence of the mature virus particles budding at the plasma membrane (Figure 2c). The viral nucleocapsids of the CHIKV measured approximately 30 nm to 40 nm in size can be seen to be lining in cytoplasm, underneath the plasma membrane (Figure 2ci). The viral nucleocapsids were seen to be expelled out of the cytoplasm through blebbing and pinching off the plasma membrane (Figure 2d), acquiring a portion of the membrane as its envelope (Figure 2di). The full mature virus was measured to be approximately 60 nm to 70 nm in size. The progression of the infection then saw an array of neatly arranged mature virus lining in rows on the peripheral of cells and in between the junction of two adjacent cells. This mode of virus release from the infected cells via budding through the plasma membrane occurred at early stages of infection and marked the first mode of viral release.

As the infection progressed, the dominant viral-induced structure was replaced by the cytopathic vacuoles type II (CPV II) complexes. This phenomenon occurred as early as 24 hours post-infection. Unlike the CPV I, these CPV II complexes were characterized by smaller vacuoles, measuring 300 nm to 400 nm in size (Figure 2e). These complexes were often observed in large number and cluster near the plasma membrane (Figure 2f). The cytoplasmic side of the CPV II vacuoles was surrounded by neatly arranged electron-dense viral nucleocapsids measuring 30 nm to 40 nm in size. These nucleocapsids can be observed to be budding into the CPV II complexes, and were thought to mature in these CPV II vacuoles. Subsequently, these CPV II vacuoles can be seen to contain full matured enveloped CHIKV viruses (Figure 2f, arrow), and the fusion of these vacuoles with the plasma membrane to release the matured virus out of the cell (Figure 2g). As such, it can be postulated that the CPV II may function as exocytosis vesicles or vacuoles for the release of the CHIKV particles at late infection. In addition, crystalline array of virus particles accumulating on the surface of the infected cells can be seen (Figure 2h). This mode of virus release through exocytosis of the CPV II complex with the fusion with the plasma membrane marked the second mode of viral release during the late stages of infection and may provide a means of rapid release of the mature viruses to the exterior of cell before cell death sets in.

**Figure 3:**
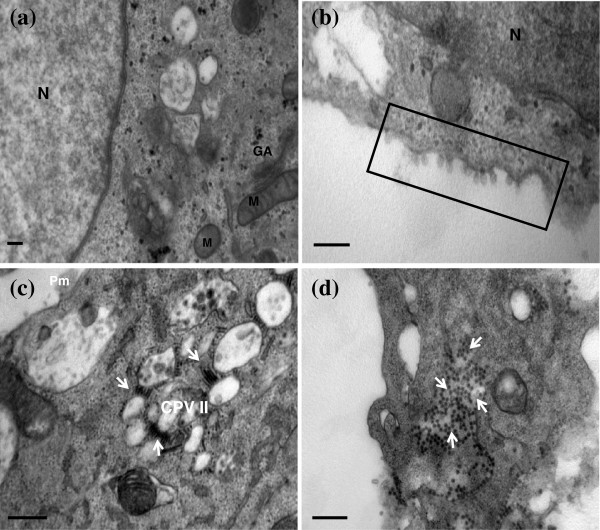
**Replication of CHIKV (IMT isolate) in HeLa cells.** (**a**) Ultrastructural analysis of mock-infected HeLa cells. Under high magnification, the cellular organelles such as the Golgi apparatus (GA) and mitochondria (M), can be seen. The bar corresponds to 0.2 μm. (**b**) Infection of CHIKV in HeLa cells. Viral-induced membranous spheres are observed to associate with the plasma membrane [boxed]. The bar corresponds to 0.2 μm. (**c**) CPV II complexes were detected at 12 hours post infection. The viral nucleocapsids (arrows) were observed to be budding in the CPV II complexes. The bar of c corresponds to 0.5 μm. (**d**) Massive accumulation of mature CHIKV (arrows) in infected cells at late infection. The bar corresponds to 0.5 μm.

CHIKV infection was also tracked in the HeLa cells with CHIKV infection. The mock-infected HeLa were characterized with typical cellular organelles as shown in Figure 3a. Generally, for the CHIKV-infected cells, the appearance of the distinct viral-induced structures remained similar to that of the infected C6/36 cells. Interestingly, CPV I complexes were no longer the predominant viral-induced structures observed at 6 hours post-infection, instead extensive membranous spheres were also observed to be lining on the plasma membrane (Figure 3b, boxed). These membranous spheres were measured to be approximately 60 nm to 80 nm and were structurally identical to the membranous spheres in the CPV I complexes (Figure 2b, arrows). It was presumed that these membranous spheres were the RNA replication site for the viral synthesis. The formation of these replication complexes was also noted to be the initial replication complexes in the vertebrate cells but not the invertebrates cells in other alphaviruses [33]. As infection progressed, matured viruses were seen to be budding off the plasma membrane as early as 8 hours post-infection (data not shown). The formation of extensive CPV II complexes can be noted as early as 12 hours post-infection. Rows of viral nucleocapsids can also be seen to be budding into the vacuoles extensively at the different planes of the cut section (Figure 3c). Similar to the infection in the C6/36 cells, the CPV II complexes were often present in cluster and located in close association to the plasma membrane. However, the number of CPV II complexes formed in HeLa cells was much more extensive than the infection in the C6/36 cells (Figure 3c). The rapid maturation and proliferation of the CHIKV virus might lead to the failure of proper exocytosis of the matured virus through fusion of the plasma membrane and resulted in the massive accumulation of matured viruses within the infected HeLa cells (Figure 3d). This may ultimately lead to the cell death of the CHIKV-infected Hela cells. However, it is also important to note that Krejbich-Trotot and workers (2011) has recently illustrated that CHIKV could also mobilize the apoptotic machinery by extrinsic and intrinsic pathways in Hela cells, a mechanism that could also be used to escape macrophages and viral host defences in vivo [34].

The re-emergence of CHIKV since 2004 has brought in much attention to this once neglected virus. This study has documented the differences in the replication kinetics of the different CHIKV isolates within the ECSA lineage. Despite the differences in the replication kinetics, the virus induced ultrastructural changes and virus morphogenesis remains highly conserved between the different CHIKV isolates of the ECSA lineage.

## Competing interests

The authors declare that they have no competing interests.

## Authors’ contributions

KCC, YWK, RTPL, MMLN, LFPN and JJHC designed this study and revised the manuscript critically; KCC and YWK carried out this study and drafted the manuscript. All of the authors read and approved the final version of this manuscript.

## Supplementary Material

Additional file 1: Table S1 Amino acid differences between the CHIKV S27 sequence (template) and three other CHIKV isolates (IMT, SGP007 and SGP011). Click here for file
