# Supplementary material for: Comparative analysis of the genome sequences and replication profiles of chikungunya virus isolates within the East, Central and South African (ECSA) lineage
Source: Virol J. 2013 May 30;10:169. doi: 10.1186/1743-422X-10-169 (PMC3679931; doi:10.1186/1743-422X-10-169)
Supplement: Additional file 1: Table S1 — Amino acid differences between the CHIKV S27 sequence (template) and three other CHIKV isolates (IMT, SGP007 and SGP011). [file 1743-422X-10-169-S1.doc]

Supplementary Table 1. Amino acid differences between the CHIKV S27 sequence (template) and three other isolates (IMT, SGP007 and SGP011).

|  | S27 | | IMT | | SGP007 | | SGP011 | |  |
| --- | --- | --- | --- | --- | --- | --- | --- | --- | --- |
| Position* | Base | Amino acid | Base | Amino acid | Base | Amino acid | Base | Amino acid | Protein |
| 60 | ACC | T | ACC | T | GCC | A | ACC | T | nsP1 |
| 137 | GCA | A | GCA | A | GTA | V | GCA | A | nsP3 |
| 524 | CGA | R | TGA | STOP | CGA | R | TGA | STOP | nsP3 |
| 75 | ACA | T | GCA | A | ACA | T | GCA | A | nsP4 |
| 563 | ATT | I | ATT | I | ACT | T | ATT | I | nsP4 |
|  |  |  |  |  |  |  |  |  |  |

|  | S27 | | IMT | | SGP007 | | SGP011 | |  |
| --- | --- | --- | --- | --- | --- | --- | --- | --- | --- |
| Position* | Base | Amino acid | Base | Amino acid | Base | Amino acid | Base | Amino acid | Protein |
| 178 | CGC | R | CGC | R | CAC | H | CGC | R | E2 |
| 226 | GCG | A | GTG | V | GCG | A | GTG | V | E1 |

*Numbers refer to the amino acid positions within the corresponding CHIKV proteins.

Red indicates a nucleotide difference resulting in an unique amino acid change in SGP7.

Blue indicates a nucleotide difference resulting in the same amino acid residue between SGP7 and S27.
